# Clinical usefulness and acceptability of small‐bowel capsule endoscopy with panoramic imaging compared with axial imaging in Japanese patients

**DOI:** 10.1002/deo2.389

**Published:** 2024-06-06

**Authors:** Issei Hirata, Akiyoshi Tsuboi, Yuka Matsubara, Akihiko Sumioka, Takeshi Takasago, Hidenori Tanaka, Ken Yamashita, Hidehiko Takigawa, Yuji Urabe, Shiro Oka

**Affiliations:** ^1^ Department of Gastroenterology Graduate School of Biomedical and Health Sciences Hiroshima University Hiroshima Japan

**Keywords:** acceptability, capsule endoscopy, diagnostic yield, obscure gastrointestinal bleeding, small‐bowel

## Abstract

**Objectives:**

We aimed to evaluate the usefulness and acceptability of CapsoCam Plus (CapsoCam) in Japanese patients.

**Methods:**

This retrospective single‐center study enrolled 930 patients with suspected small‐bowel bleeding (SSBB) who underwent capsule endoscopy. Thirty‐three patients using CapsoCam and PillCam SB3 (SB3) were matched using propensity score matching. The diagnostic yield and the acceptability of CapsoCam were evaluated.

**Results:**

There was no SSBB case where capsule endoscopy was performed within 48 h of bleeding. CapsoCam had a significantly higher observation rate of the entire small bowel (97% vs. 73%, *p* = 0.006) and Vater's papilla (82% vs. 15%, *p* < 0.001) than SB3. The reading time of CapsoCam was significantly longer than that of SB3 (30 vs. 25 min, *p* < 0.001), and CapsoCam's time from the capsule endoscopy swallowing to read completion was longer than that of SB3 (37 vs. 12 h, *p* < 0.001). The two groups showed no difference in the capsule endoscopy findings according to the P classification. Notably, 85% of the patients using CapsoCam reported examination distress as “not at all” or “almost not,” and 94% reported swallowing difficulty as “very easy” or “easy.”

**Conclusions:**

CapsoCam took time to read; however, it is a well‐tolerated examination with a high observation rate of Vater's papilla and entire small‐bowel mucosa. Detectability of bleeding sources was comparable in both modalities for cases of occult SSBB and overt SSBB more than 48 h after bleeding. CapsoCam is a useful modality for patients with SSBB.

## INTRODUCTION

The diagnosis and endoscopic treatment of small‐bowel disorders have recently become easier with the widespread use of small‐bowel endoscopies in daily clinical practice. Small‐bowel bleeding accounts for 5% of all gastrointestinal bleeding,^1^ making it a common small‐bowel disorder. Small‐bowel bleeding is diagnosed by overt hemorrhage with bloody stools or hematemesis or occult hemorrhage with chronic anemia. In Japan, the term “obscure gastrointestinal bleeding (OGIB)” is defined as gastrointestinal bleeding of unknown origin with no apparent bleeding source, as observed using esophagogastroduodenoscopy and colonoscopy.^2^ However, in Europe and the United States, the term “OGIB” should be used only for patients with unidentifiable bleeding sources in the digestive tract using small‐bowel endoscopy.^3^, ^4^ Capsule endoscopy (CE), with its widespread use, is positioned as the first‐line modality for small‐bowel bleeding because of its low invasiveness and high diagnostic ability.^3^, ^4^

Since the first CE report by Iddan et al.^5^ in 2000, there have been improvements in the endoscopic equipment, and various models have been developed. PillCam SB (Covidien) was the earliest developed CE device,^6^ and others, for example, the EndoCapsule (Olympus),^7^ MiroCam (IntroMedic),^8^ and the OMOM capsule (Jinshan Science and Technology) are also in global use. In 2013, Friedrich et al.^9^ reported that the novel endoscopic device (CapsoCam SV1; CapsoVision) had four cameras for 360° panoramic imaging. CapsoCam Plus (CapsoCam; CapsoVision) is a third‐generation CE device with a panoramic view, available since January 2021 for clinical use in patients with suspected small‐bowel bleeding (SSBB) in Japan. CapsoCam can perform 360° panoramic imaging, and the captured data are stored in an internal flash memory, eliminating the need for a portable receiver. In Japan, conventional axial‐view type CE devices are contraindicated in patients with implanted pacemakers or cardioverter defibrillators due to radiofrequency external transmission to an external receiver, but CapsoCam can be used.

Two reports currently compare the diagnostic yield for patients with OGIB using axial‐view type CE with the previous generation of CapsoCam.^10^, ^11^ Notably, both reports showed no significant differences in the diagnostic yield. However, CapsoCam may have an improved diagnostic yield compared with the previous generation owing to a modified CMOS image sensor, flash memory, and improved image quality. A comparative study for patients with celiac disease, evaluating the diagnostic capabilities of CapsoCam and PillCam SB3 (SB3), revealed a comparable diagnostic yield in both modalities.^12^ Here, we compared patients using CapsoCam and SB3 among the patients with SSBB to evaluate CapsoCam's usefulness. We also evaluated the acceptability of CapsoCam using a questionnaire.

## METHODS

### Patients

**FIGURE 1:**
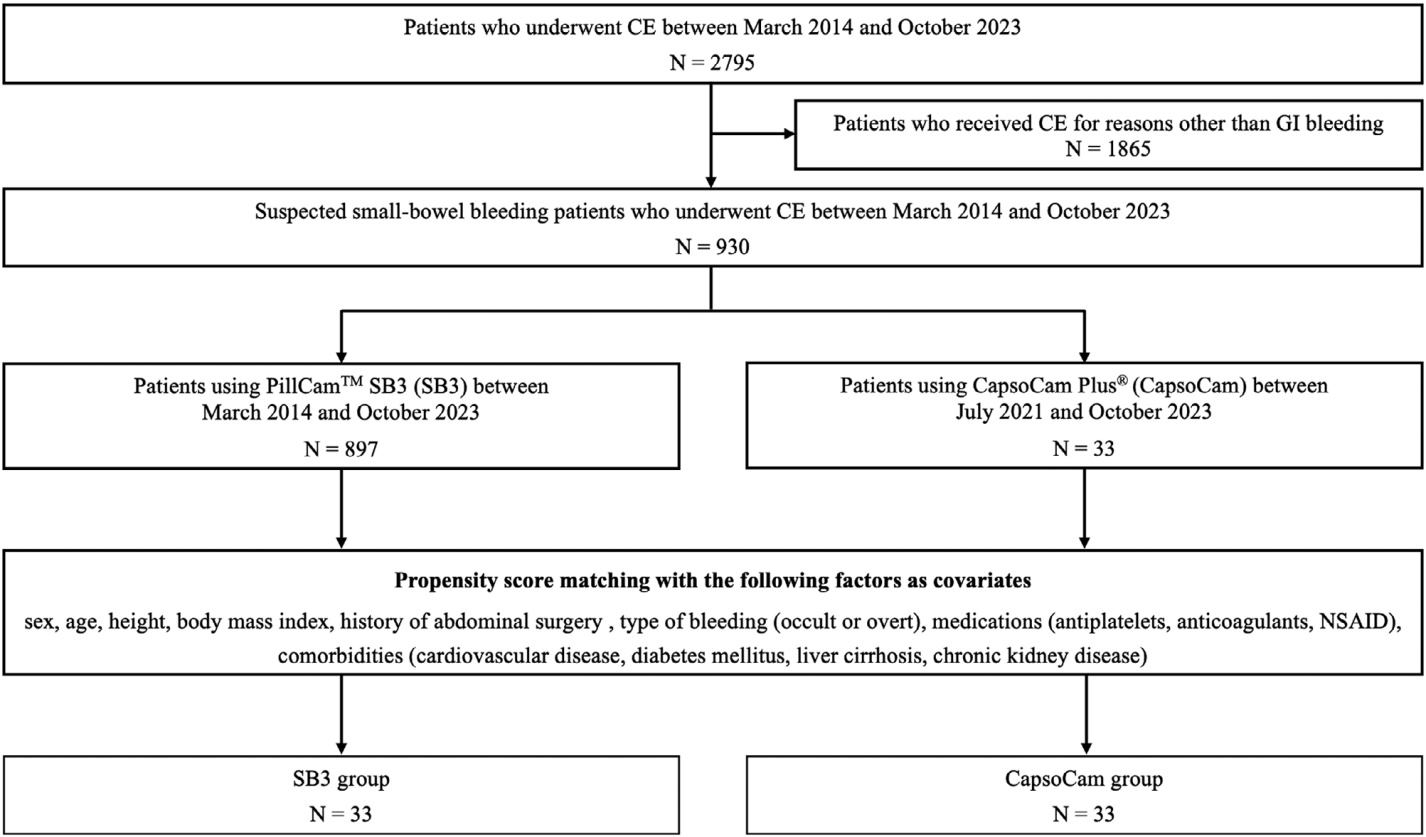
Study flowchart. Abbreviation: CE, capsule endoscopy.

We retrospectively examined 2795 consecutive patients who underwent CE at Hiroshima University Hospital between March 2014 and October 2023. Figure 1 shows the study's flowchart. Among the eligible patients, 930 underwent CE for SSBB, with 897 patients undergoing SB3. CapsoCam has been used in 33 patients since July 2021. Propensity score matching was conducted with covariates to minimize bias based on patient backgrounds.

The study was conducted following the principles of the Declaration of Helsinki. All patients provided written informed consent. This study was approved by the Hiroshima University Hospital's Institutional Review Board (registration number: E2022‐0309).

### CE procedures

All patients underwent CE using SB3 between April 2014 and June 2021. Two expert endoscopists for small‐bowel CE selected the SB3 or CapsoCam between July 2021 and October 2023 based on their judgment. The same preparation protocols were used in both groups (overnight fasting without any other preparation). Patients were administered clear liquids and a light meal 2 and 4 h after swallowing the capsule. The protocols to resume drinking and eating after the examination were the same for both groups. For using SB3, the sensor array and recording device were removed 8 h after, and the obtained images were analyzed using the Rapid Reader 6.5 software (Quest Software) or RAPID 8 workstation (Covidien). Patients who used CapsoCam recovered the CE device using a Capsule Recovery Kit and brought the device to our hospital. After cleaning the capsule, the images were extracted using CapsoAccess (CapsoVision) and evaluated using CapsoView (CapsoVision). Two experienced physicians (Akiyoshi Tsuboi and Shiro Oka) read the images extracted from the CE device; each physician had previously interpreted > 100 videos of CE (SB2 or SB3) and was certified by the Japanese Society for Capsule Endoscopy. The two endoscopists made the diagnosis based on CE findings. In disagreement, a consensus on the diagnosis was reached through discussion. In principle, double‐balloon endoscopy (DBE) was performed for patients with P2 CE findings.

### Evaluation

**FIGURE 2:**
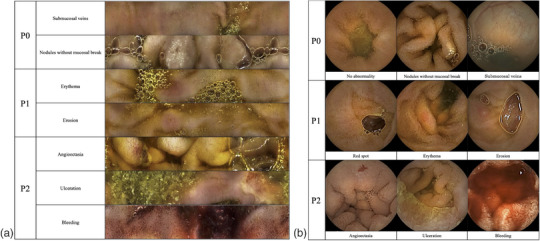
Capsule endoscopic images classified by P classification for each modality. (a) CapsoCam Plus and (b) PillCam SB3.

The following patient data were obtained from electronic medical charts: sex, age, height, body mass index, history of abdominal surgery, bleeding type (occult or overt), medications (antiplatelet, anticoagulant, and non‐steroidal anti‐inflammatory drug [NSAID]), comorbidities (cardiovascular disease [CVD], diabetes mellitus, liver cirrhosis [LC], and chronic kidney disease [CKD]), and previous CE examination. The variable data for CE results included the availability of image analysis (capsule retrieval rate in the CapsoCam group), rate of entire small‐bowel observation, rate of Vater's papilla observation, each segment's transit times, reading time, time from CE device swallowing to excretion, number of bowel movements until CE device excretion, time from CE device swallowing to the completion of reading, and CE findings. The CE findings were classified into three groups based on Saurin et al.’s classification^13^ Figure 2 shows an example of P classification, where P0 was defined as lesions with no bleeding potential; P1 was defined as lesions with an uncertain bleeding potential; and P2 was defined as lesions with a high bleeding potential or confirmed bleeding.

Additionally, the acceptability of the CE examination was assessed through a questionnaire using a face scale to subjectively evaluate the examination's discomfort and capsule swallowing difficulty levels in patients using CapsoCam. A similar questionnaire was used for patients previously examined using the SB3 to obtain subjective evaluations of the same items. The study's primary outcome was CE examination results between the two groups after propensity score matching. The secondary outcome was CE's acceptability based on the questionnaire results.

### Statistical analysis

Non‐normally distributed data are reported as medians and interquartile ranges (IQRs). Comparisons were performed using the chi‐square, Fisher's exact, and Mann–Whitney U tests for categorical data. Statistical significance was set at *p* < 0.05. The JMP Pro 16 software (SAS) was used for statistical analyses.

## RESULTS

Among 930 consecutive patients with SSBB who underwent CE, 33 patients (4%) used CapsoCam, and 897 patients (96%) used SB3. Table S1 presents the baseline patient characteristics by group. Three patients (9%) had implanted pacemakers, and one (3%) had an implanted cardioverter defibrillator in the CapsoCam group. In the CapsoCam group, 16 patients (48%) had a history of using SB3.

Based on propensity score matching, 33 patients were extracted from each group, and both groups showed no significant differences in baseline characteristics (Table 1). All extracted patients were able to swallow CE, no endoscopic assistance was provided during CE examinations in both groups of study patients. Table 2 presents the results of both groups’ CE examinations. In the CapsoCam group, all patients could retrieve CE images without equipment or other interpretation problems. The observation rate of the entire small bowel was significantly higher in the CapsoCam group than in the SB3 group (97% vs. 73%, *p* = 0.006). The Vater's papilla's observation rate was significantly higher in the CapsoCam group than in the SB3 group (82% vs. 15%, *p* < 0.001). In the CapsoCam group, the median time from swallowing to capsule retrieval was 24 h (IQR 13–49) with a median of three bowel movements (IQR 1–5). The two groups showed no statistically significant difference in CE findings according to P classification (*p* = 0.757). All patients with P2 CE findings underwent DBE, and the responsible lesions were detected by DBE in all cases. In the CapsoCam group, six patients (18%) had P2 findings, and three patients (9%) with angioectasia were treated with endoscopic hemostasis using DBE. In the SB3 group, seven patients (21%) had P2 findings, two patients (6%) had angioectasia, one patient (3%) with tumor bleeding was treated with DBE, and one patient (3%) with a tumor was treated surgically.

**TABLE 1:** Baseline characteristics of enrolled patients after propensity score matching.

Variables	CapsoCam Plus *n* = 33	PillCam SB3 *n* = 33	*p*‐value
Sex			1.000
Male	20 (61)	20 (61)	
Female	13 (39)	13 (39)	
Age (years), median (IQR)	71 (56–75)	67 (53–75)	0.521
Height (cm), median (IQR)	164 (159–169)	163 (157–168)	0.581
Body mass index (kg/m^2^), median (IQR)	22.3 (19.8–25.3)	21.9 (19.6–24.5)	0.798
History of abdominal surgery	12 (36)	09 (27)	0.428
Type of gastrointestinal bleeding			
Overt	13 (39)	12 (36)	0.800
Examination within 48 h of bleeding	0 (00)	00 (0)	1.000
Occult	20 (61)	21 (64)	0.800
Medications^†^			
Antiplatelets	07 (21)	05 (15)	0.523
Anticoagulants	05 (15)	04 (12)	0.720
Non‐steroidal anti‐inflammatory drug	03 (9)	05 (15)	0.451
Comorbidities^†^			
Cardiovascular disease	11 (33)	09 (27)	0.592
Diabetes mellitus	06 (18)	07 (21)	0.757
Liver cirrhosis	03 (9)	01 (3)	0.302
Chronic kidney disease	02 (6)	02 (6)	1.000

^†^ duplication. (%)

Abbreviation: IQR, interquartile range.

**TABLE 2:** Results associated with the capsule endoscopy.

Variables	CapsoCam Plus *n* = 33	PillCam SB3 *n* = 33	*p*‐value
Available for CE reading	33 (100)	33 (100)	1.000
Observation of the entire small bowel	32 (97)	24 (73)	0.006
Observation of Vater's papillae	27 (82)	05 (15)	< 0.001
Time of transit			
Esophagus (s), median (IQR)	5 (3–14)	3 (2–19)	0.439
Stomach (min), median (IQR)	22 (13–57)	20 (10–64)	0.753
Small‐bowel (min), median (IQR)	251 (215–340)	277 (192–344)	0.817
Time from CE swallowing to excretion (h)	24 (13–49)	‐	‐
Number of bowel movements until CE excretion	3 (1–5)	‐	‐
Complications	00 (00)	00 (0)	1.000
Retention	00 (00)	00 (0)	1.000
CE findings (P classification)			0.757
P0/ P1	27 (82)	26 (79)	
P2	06 (18)	07 (21)	
Angioectasia	3 (50)	2 (29)	
Ulceration	2 (33)	1 (14)	
Bleeding	1 (17)	2 (29)	
Tumor with bleeding	0 (0)	2 (29)	

(%)

Abbreviations: CE, capsule endoscopy; IQR, interquartile range.

**FIGURE 3:**
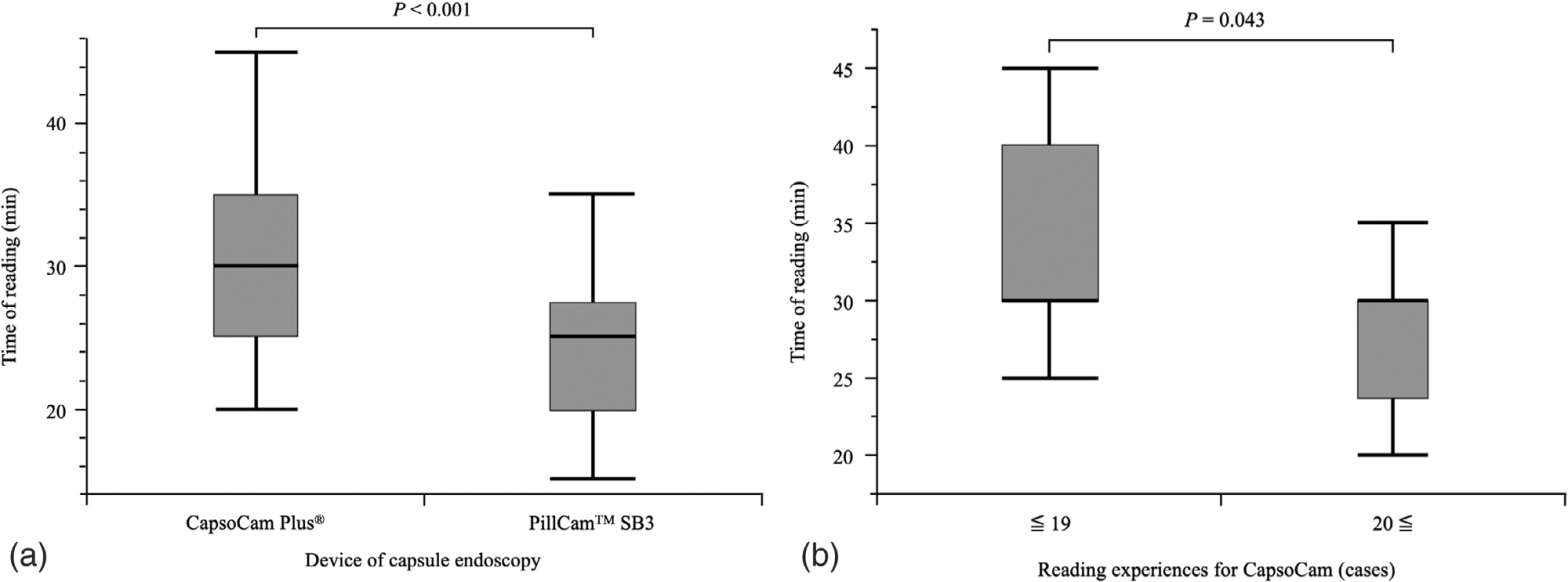
Comparison of reading times for capsule endoscopy. (a) According to each device and (b) according to the number of reading experiences for CapsoCam Plus.

**FIGURE 4:**
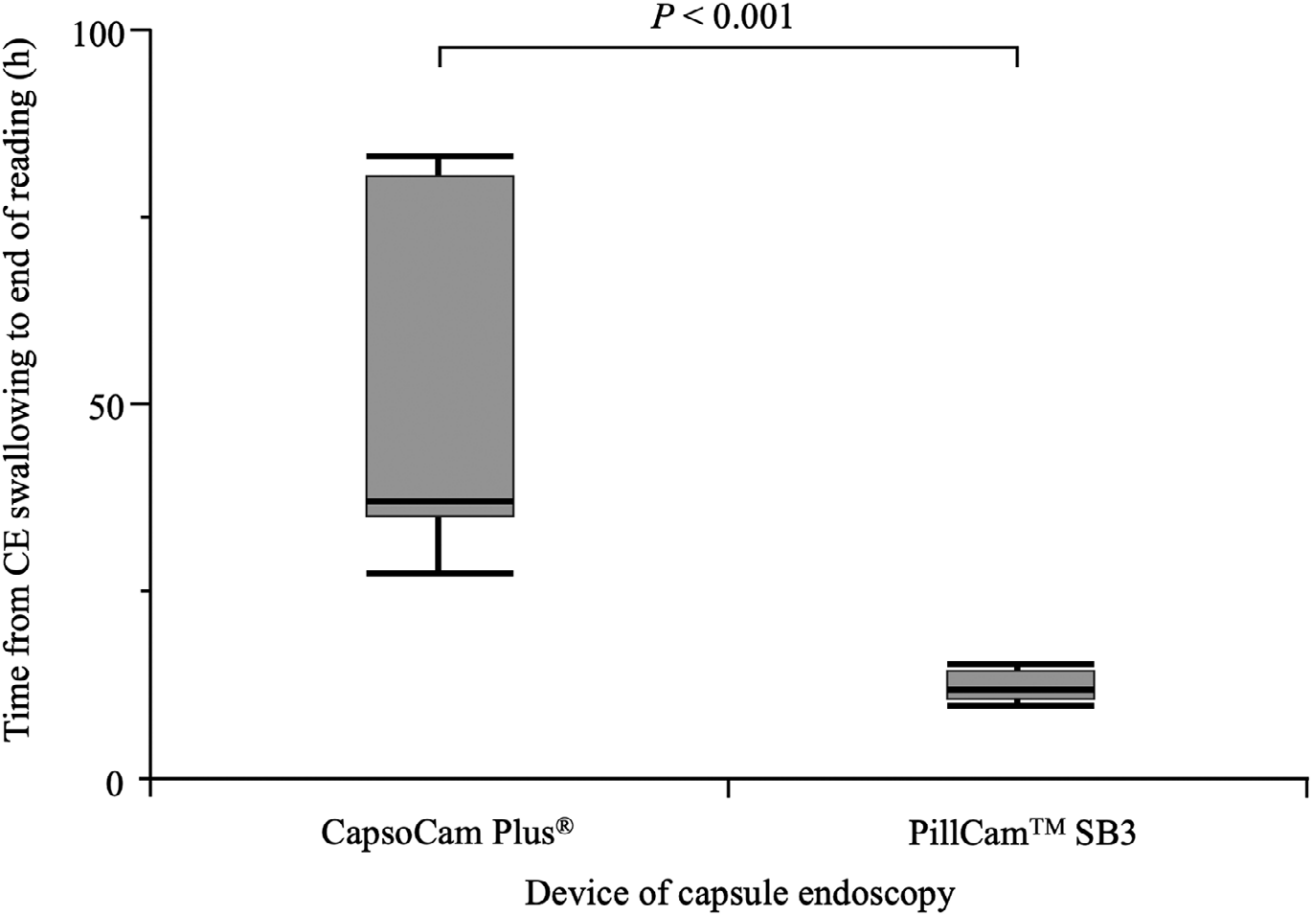
Time from capsule swallow to end of reading for each capsule endoscopy device.

Figure 3 shows the reading time analysis. The median reading times were 30 min (IQR 25–35) in the CapsoCam group and 25 min (IQR 20–28) in the SB3 group (*p* < 0.001; Figure 3A). In a subgroup analysis of the CapsoCam group based on physician reading experience, the reading time was significantly shorter when the CapsoCam reading experience was 20 cases or more compared to 19 cases or fewer (Figure 3B). The CapsoCam group had a median time of 37 h (IQR 35–81) from swallowing the capsule to completing reading images, whereas that of the SB3 group was 12 h (IQR 11–15; Figure 4). The CapsoCam examination required a significantly longer time to complete the reading from swallowing the CE device than the SB3 due to the waiting period for CE device excretion.

**FIGURE 5:**
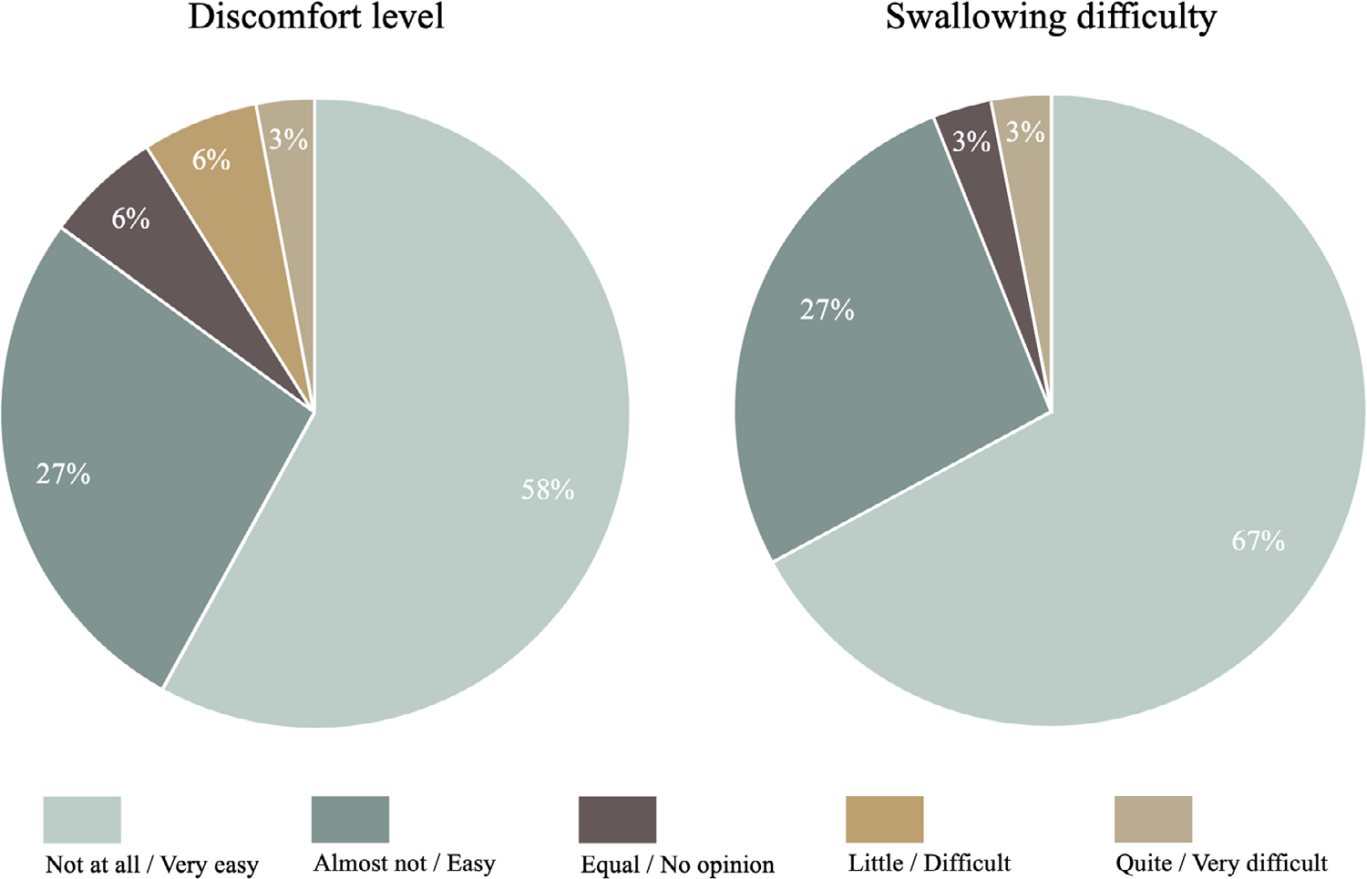
Examination discomfort level and capsule swallowing difficulty level of CapsoCam Plus.

Figure 5 shows the acceptability results for patients using CapsoCam. Regarding the CE examination's discomfort level, 28 patients (85%) answered with “not at all” or “almost not.” Conversely, three patients (9%) responded with “little” or “quite” discomfort because of difficulty in capsule retrieval and 2) mental burden due to constipation, requiring 1 week for capsule excretion. Regarding the capsule swallowing difficulty, 31 patients (94%) answered with “very easy” or “easy.” Only one patient (3%) answered with “difficult.”

**FIGURE 6:**
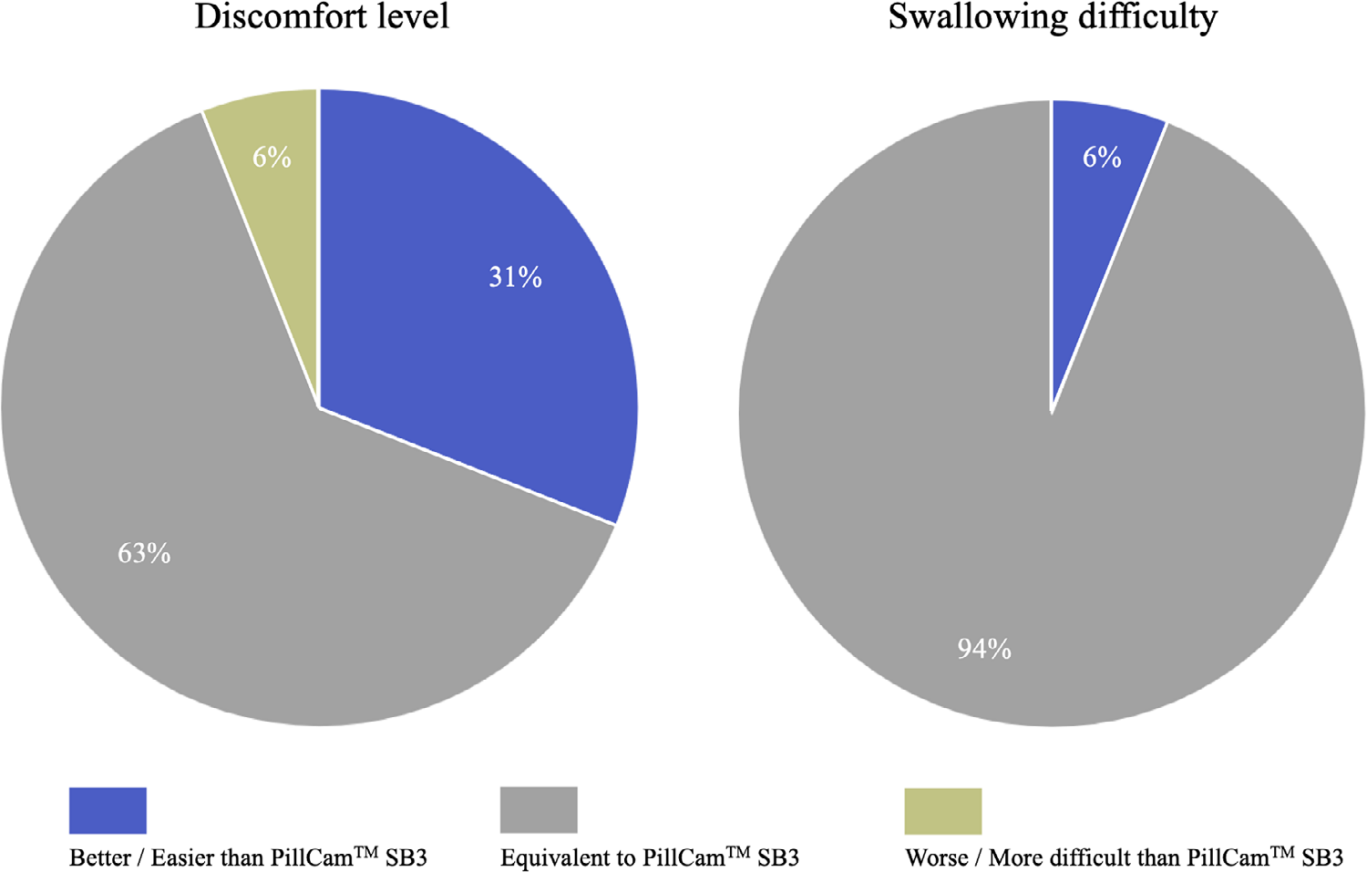
Comparison of the examination discomfort level and the swallowing difficulty level of CapsoCam Plus and PillCam SB3.

Sixteen patients were examined using both modalities. Five (31%) reported lower discomfort with CapsoCam than with SB3 (Figure 6) because of the absence of a sensor array and recording device. However, 15 patients (94%) reported no difference between both devices regarding the swallowing difficulty.

## DISCUSSION

Our study revealed that a third‐generation CE device with a panoramic view had a high observation rate of Vater's papilla and entire small‐bowel mucosa, and a high acceptability for patients with SSBB. For the detectability of the bleeding source, the panoramic‐view CE showed no significant difference in the P classification compared to the axial‐view CE. Although the panoramic‐view CE is highly acceptable to the examinee, the reading time for the panoramic‐view CE was significantly longer than that for the axial‐view CE. Therefore, the panoramic view of CE is a burdensome examination for the reader. However, it was estimated that the reading time would decrease as the number of readings increased. To our knowledge, there are no reports comparing the reading outcomes and patient acceptability between third‐generation panoramic‐view CE and axial‐view CE.

With the development of endoscopic devices, various types of CE devices have become available worldwide. Supplementary Table S2 presents a comparison of CapsoCam and SB3 features. In a recent meta‐analysis,^14^ akin to our study, no significant differences in diagnostic yield among different types of CE were reported between CapsoCam and SB3. The diagnostic yield of P2 findings was 18% and 21% in the CapsoCam and SB3 groups, respectively, which were comparable in terms of picking up P2 findings. The diagnostic yield for CE in patients with OGIB was 55%–62%.^15^, ^16^, ^17^ Our study's rate of P2 findings was lower than that reported previously. The factors associated with positive CE findings in patients with OGIB were old age, low hemoglobin levels, massive transfusion, overt OGIB, NSAID use, and CE within 48 h of the final bleeding episode.^4^, ^18^, ^19^, ^20^ No patient had undergone CE within 48 h of overt bleeding here. The rate of patients with occult OGIB was reported as 20.4%–41.4%.^19^, ^20^, ^21^, ^22^ This study's rate of occult bleeding was approximately 60%, higher than that in previous reports. We reported that the detection rate of the bleeding source for patients with occult OGIB was 27%.^23^ Regarding comorbidities, LC, CKD, and CVD (severe aortic stenosis) were risk factors for small‐bowel angioectasia^24^, ^25^, ^26^; however, the number of patients with these factors in our study was limited. It was also not used in cases of suspected intestinal stenosis due to the absence of a patency capsule. These patient backgrounds may have influenced our study results.

Similar to previous reports,^9^, ^27^, ^28^ the observation rates for the entire small bowel and Vater's papilla were 97% and 82%, respectively, significantly higher than those of conventional CE. The factor affecting the entire small‐bowel observation is thought to have a longer battery life than the SB3. The battery lives of SB3 and CapsoCam were estimated at approximately 12 and 15 h, respectively. The longer battery life for CapsoCam could be due to no need for external image transmission and the existence of the Smart Motion function (which reduces image capture by not recording if the current frame matches the previous frame). Additionally, at our hospital, the external receiver of the SB3 was retrieved 8 h after CE swallowing, which may have affected the results. The high observation rate of the Vater's papilla in the CapsoCam group is likely because of the 360° panoramic view and the high frame rate of CapsoCam compared with SB3. In examinations using SB3, the risk of oversight has been reported in the proximal jejunum, where the capsule moves quickly.^29^ The present study indicated that CapsoCam may help detect proximal jejunal lesions. However, the lack of an external receiver and the absence of a real‐time viewer remain a concern. In cases of hypotonic gastrointestinal peristalsis, stagnation may occur in the esophagus and stomach.^30^, ^31^ Importantly, it is not possible to switch to capsule delivery by verifying the real‐time viewer in such cases.

The CapsoCam group required a significantly longer time to read images than the SB3 group, which is consistent with previous results.^10^, ^11^ We attribute this to the learning curve associated with interpreting panoramic‐view CE. Given the volume of images in CE, the process can be arduous for physicians, particularly trainees, resulting in increased time and psychological stress.^32^ For the reading time of axial‐view CE, the trainee's reading time was longer than that of the experts.^33^ Here, a significant reduction in interpretation time was observed when comparing cases with fewer than 19 interpretations to those with 20 or more. In the case of CapsoCam, we expected to reduce the reading time depending on the physician's proficiency as with the axial‐view CE. Furthermore, the time from swallowing CE to the end of reading was significantly longer in the CapsoCam group than in the SB3 group. This result is affected by the necessity of waiting for CE excretion to read CapsoCam. Short intervals between CE and DBE are more effective for diagnosis and treatment.^34^ Generally, physicians decide whether to perform DBE according to the results of CE in SSBB cases. Therefore, we consider that CapsoCam should be carefully used for cases of overt SSBB in the early period from the last bleeding, as it takes time to read.

CapsoCam had almost the same short diameter but a longer long diameter than SB3. However, the answers to the question about the difficulty swallowing the CE device for CapsoCam were mostly the same as those for SB3. Regarding the discomfort level, eliminating the portable receiver made the CapsoCam examination acceptable in many cases. The severe acute respiratory syndrome coronavirus 2 pandemic has recently spread globally, and the usefulness of CE devices at home or work using CapsoCam has been reported.^28^ Unlike conventional CE, CapsoCam examinations do not necessitate attaching a sensor array to the body, making it convenient for self‐examination at home or work.

This study has some limitations. First, being a retrospective single‐center study, it had a limited number of CapsoCam cases, possibly affecting result generalizability. Secondly, there was selection bias with CapsoCam usage, impacting diagnostic performance. Thirdly, there was a considerable gap between CapsoCam and SB3 usage periods in many cases. Fourthly, DBE examinations were not performed in all cases, and without a gold standard for diagnosis, true diagnostic accuracy remains unknown. Fifth, there were differences in CE examination procedures in each modality. This difference may have affected the difference in the rate of entire small‐bowel observation. Sixth, in overt SSBB, examination within 48 h of onset was useful in detecting the source of the bleeding, but none of the cases in this study were examined with CE at that time. Large‐scale prospective studies across multiple medical institutions, including cases with early examination from the last bleeding, are needed to address these limitations, enhancing reliability, and generalizability, and providing comprehensive insights.

In conclusion, CapsoCam offers comparable detectability of bleeding sources for cases of occult SSBB and overt SSBB more than 48 h after bleeding. Additionally, CapsoCam had superior visualization of the entire small bowel and Vater's papillae compared to SB3, with excellent patient tolerance.

## CONFLICT OF INTEREST STATEMENT

The authors declare no conflict of interest.

## Supporting information

Supplementary Tables 1 and 2
