# Supplementary material for: Clinical usefulness and acceptability of small‐bowel capsule endoscopy with panoramic imaging compared with axial imaging in Japanese patients
Source: DEN Open. 2024 Jun 6;5(1):e389. doi: 10.1002/deo2.389 (PMC11154819; doi:10.1002/deo2.389)
Supplement: Supplementary file 1 — Supplementary Tables 1 and 2 [file DEO2-5-e389-s001.docx]

**Supplementary Table 1. Baseline characteristics of enrolled patients before propensity score matching**

| Variables | | | CapsoCam Plus^®︎^  n = 33 | | PillCam^TM^ SB3  n = 897 | | *P*-value |
| --- | --- | --- | --- | --- | --- | --- | --- |
| Sex | | |  | |  | | 0.697 |
|  | Male | | 20 (61) | | 513 (57) | |  |
|  | Female | | 13 (39) | | 384 (43) | |  |
| Age (years), median (IQR) | | | 71 (56–75) | | 71 (60–78) | | 0.320 |
| Height (cm), median (IQR) | | | 164 (159–169) | | 159 (153–166) | | 0.002 |
| Body mass index (kg/m^2^), median (IQR) | | | 22.3 (19.8–25.3) | | 21.5 (19.2–24.5) | | 0.200 |
| History of abdominal surgery | | | 12 (36) | | 377 (42) | | 0.517 |
| Type of gastrointestinal bleeding | | |  | |  | | 0.019 |
|  | Overt | | 13 (39) | | 537 (60) | |  |
|  | Occult | | 20 (61) | | 360 (40) | |  |
| Medications^†^ | | |  | |  | |  |
|  | Antiplatelets | | 07 (21) | | 192 (21) | | 0.979 |
|  | Anticoagulants | | 05 (15) | | 161 (18) | | 0.632 |
|  | Non-Steroidal Anti-Inflammatory Drug | | 03 (09) | | 119 (13) | | 0.485 |
| Comorbidities^†^ | | |  | |  | |  |
|  | Cardiovascular disease | | 11 (33) | | 241 (27) | | 0.412 |
|  |  | Pacemaker implantation |  | 4 (12) |  | 0 (00) | < 0.001 |
|  |  | ICD implantation |  | 1 (03) |  | 0 (00) | < 0.001 |
|  | Diabetes mellitus | | 06 (18) | | 198 (22) | | 0.596 |
|  | Liver cirrhosis | | 03 (09) | | 231 (26) | | 0.030 |
|  | Chronic kidney disease | | 02 (06) | | 150 (17) | | 0.104 |
| History of using PillCam^TM^ SB3 | | | 16 (48) | | - | | - |
| †duplication  ICD: implantable cardioverter defibrillator | | | | | | | (%) |

**Supplementary Table 2. Feature comparison of CapsoCam Plus^®︎^ and PillCam^TM^ SB3**

|  | CapsoCam Plus^®︎^ | PillCam^TM^ SB3 |
| --- | --- | --- |
| Lemgth (mm) | 31 | 26.2 |
| Diameter (mm) | 11 | 11.4 |
| Weight (g) | 4.0 | 3.0 |
| Battery life (h) | 15 | 12 |
| Frame rate (fps) | 12–20 | 2–6 |
| LEDs | 16 | 4 |
| Field of View (degree) | 360 | 156 |
| Transmission | Onboard Storage System | Radiofrequency |
